# COVID-19-Related Free Telephone Consultations by Public Health Nurses

**DOI:** 10.1093/geroni/igaa057.3444

**Published:** 2020-12-16

**Authors:** Yuka Sumikawa, Chikako Honda, Kyoko Yoshioka-Maeda, Riho Iwasaki-Motegi, Noriko Yamamoto-Mitani

**Affiliations:** 1 Graduate School of Medicine, The University of Tokyo, Tokyo, Japan; 2 National Institute of Public Health, Wako City, Japan

## Abstract

Public health centers are located in each municipality in Japan and are responsible for infectious disease control including COVID-19. Public health nurses (PHNs) are stationed at the centers and work at the forefront, covering a variety of services from individual consultations to hospital escort for those tested positive. Starting January, PHNs at A city (population approx. 210,000) established a free telephone consultation hotline for COVID-19. This study aims to review the PHNs’ telephone consultations during the first wave of COVID-19. The number of calls were aggregated weekly and their time-trend was examined. The study was approved by the University of Tokyo Ethics Review Board. During the first wave between January and May, there were 3,242 calls, with the highest number of calls (n=491/week) in the second week of April. At this point the regular PHNs were not enough to meet the hightened needs of consultations and PHNs from other departments were temporalily transferred for support. The number of consultation calls fluctuated weekly. The increase of calls seemed to preceed the increase of positive cases by one week. We consider that the call may be an initial action of those who suspected possible infection, and the consultation by the PHN might have led them to proper clinic visits and PCR testing. Telephone consultation is an easy tool to use for general public, especially older persons. Having health professionals respond directly to calls may have had the advantage of providing appropriate guidance for infection control and PCR testing and mental support.

